# Maternal stress during pregnancy alters circulating small extracellular vesicles and enhances their targeting to the placenta and fetus

**DOI:** 10.1186/s40659-024-00548-4

**Published:** 2024-09-28

**Authors:** Mario Sánchez-Rubio, Lorena Abarzúa-Catalán, Ana del Valle, Maxs Méndez-Ruette, Natalia Salazar, Jacinta Sigala, Soledad Sandoval, María Inés Godoy, Alejandro Luarte, Lara J. Monteiro, Roberto Romero, Mahesh A. Choolani, Úrsula Wyneken, Sebastián E. Illanes, Luis Federico Bátiz

**Affiliations:** 1 Research Program in Neuroscience, Center for Biomedical Research and Innovation (CiiB), Universidad de los Andes, Santiago, Chile; 2 PhD Program in Biomedicine, Facultad de Medicina, Universidad de los Andes, Santiago, Chile; 3 IMPACT, Center of Interventional Medicine for Precision and Advanced Cellular Therapy, Santiago, Chile; 4 School of Medicine, Facultad de Medicina, Universidad de los Andes, Santiago, Chile; 5 Department of Educational Assessment, Measurement, and Registry, Universidad de Chile, Santiago, Chile; 6 Research Program in Biology of Reproduction, Center for Biomedical Research and Innovation (CiiB), Universidad de los Andes, Santiago, Chile; 7 Pregnancy Research Branch, Division of Obstetrics and Maternal-Fetal Medicine, Division of Intramural Research, Eunice Kennedy Shriver National Institute of Child Health and Human Development, National Institutes of Health, United States Department of Health and Human Services (NICHD/NIH/DHHS), Bethesda, Detroit, and Maryland, Michigan USA; 8 Department of Obstetrics and Gynecology, University of Michigan, Ann Arbor, MI USA; 9 Department of Epidemiology and Biostatistics, Michigan State University, East Lansing, MI USA; 10 Division of Maternal Fetal Medicine, Department of Obstetrics and Gynecology, Yong Loo Lin School of Medicine, National University of Singapore, Singapore, Singapore; 11 Department of Obstetrics and Gynecology, School of Medicine, Facultad de Medicina, Universidad de los Andes, Santiago, Chile; 12 Centro de Investigación e Innovación Biomédica (CiiB), Facultad de Medicina, Universidad de los Andes, Mons. Álvaro del Portillo 12455, Las Condes, Santiago, Chile

**Keywords:** Prenatal stress, Restraint, Sex-bias, Placenta, Fetus, Biodistribution, Exosomes

## Abstract

**Background:**

Maternal psychological distress during pregnancy can negatively impact fetal development, resulting in long-lasting consequences for the offspring. These effects show a sex bias. The mechanisms whereby prenatal stress induces functional and/or structural changes in the placental-fetal unit remain poorly understood. Maternal circulating small extracellular vesicles (sEVs) are good candidates to act as “stress signals” in mother-to-fetus communication. Using a repetitive restraint-based rat model of prenatal stress, we examined circulating maternal sEVs under stress conditions and tested whether they could target placental-fetal tissues.

**Results:**

Our mild chronic maternal stress during pregnancy paradigm induced anhedonic-like behavior in pregnant dams and led to intrauterine growth restriction (IUGR), particularly in male fetuses and placentas. The concentration and cargo of maternal circulating sEVs changed under stress conditions. Specifically, there was a significant reduction in neuron-enriched proteins and a significant increase in astrocyte-enriched proteins in blood-borne sEVs from stressed dams. To study the effect of repetitive restraint stress on the biodistribution of maternal circulating sEVs in the fetoplacental unit, sEVs from pregnant dams exposed to stress or control protocol were labeled with DiR fluorescent die and injected into pregnant females previously exposed to control or stress protocol. Remarkably, maternal circulating sEVs target placental/fetal tissues and, under stress conditions, fetal tissues are more receptive to sEVs.

**Conclusion:**

Our results suggest that maternal circulating sEVs can act as novel mediators/modulators of mother-to-fetus stress communication. Further studies are needed to identify placental/fetal cellular targets of maternal sEVs and characterize their contribution to stress-induced sex-specific placental and fetal changes.

**Supplementary Information:**

The online version contains supplementary material available at 10.1186/s40659-024-00548-4.

## Background

Unfavorable prenatal environments, such as maternal psychological distress -comprising perceived stress and depressive and anxiety symptoms- during pregnancy (also known as prenatal stress (PS)), can profoundly affect fetal development and predispose the offspring to long-lasting adverse neurodevelopmental outcomes [1–7]. Given that the overall prevalence of psychological distress in pregnant women is higher than in the general population [8–12], understanding the biological mechanisms underlying maternal-fetal communication under stress conditions is critical for designing ways of preventing or reducing PS-related adverse outcomes.

Prenatal stress is associated with a range of adverse perinatal outcomes, including reduced fetal growth [13], spontaneous abortion, preterm delivery, and preeclampsia [14–16], and is also linked to poor neurodevelopmental outcomes in the offspring, such as behavioral problems [17, 18], cognitive deficits [19–24], lower academic performance [25], and increased risks of neurodevelopmental disorders such as attention deficit/hyperactivity disorder (ADHD), autism spectrum disorder (ASD), and schizophrenia [17–19, 21, 23, 26–28]. Structural and functional brain changes, such as reduced gray matter volume [29], enlarged amygdala [30, 31], and decreased hippocampal volume [31], may underlie PS-related neurodevelopmental outcomes. Interestingly, PS-dependent neuropathological and/or clinical effects can vary based on the complex interplay between stressor type, genetic background, and gestational period of exposure [6, 32]. Furthermore, several studies in humans and animal models have revealed sexually dimorphic responses to PS [33–40]. In this context, the placenta, with its intrinsic sexual dimorphism, may play a critical role in mediating, modulating, or potentiating mother-to-fetus stress-transferring signals with a sexual bias [41].

Despite the associations between maternal psychological distress and neurodevelopmental dysfunctions in the offspring, the mechanisms whereby PS induces functional and/or structural changes in the placenta and fetus remain poorly understood [41]. Small extracellular vesicles (sEVs) have emerged as complex mediators of intercellular communication, transporting proteins, lipids, and RNAs between distant cells [42, 43]. These vesicles, which, according to their size and origin, are classified in exosomes, microvesicles, and apoptotic bodies, can modify the physiology of recipient cells [42–45]. Our previous work using two restraint-based stress protocols in male rats demonstrated that stress-responsive brain cells (astrocytes) could undergo stress-induced morphological changes and modify the cargo of sEVs secreted into the bloodstream, potentially acting as stress signals to peripheral tissues [46, 47]. Remarkably, during pregnancy, the number of circulating sEVs increases [48, 49], and emerging evidence suggests that these maternal sEVs can target placental and fetal tissues [50–52]. This study aims to explore how PS affects maternal circulating sEVs, potentially revealing novel mechanisms of maternal-fetal communication and stress-induced fetal programming.

Animal models of PS vary widely, but the repetitive restraint stress protocol (i.e., daily exposure to a period of movement restriction) is commonly used for its simplicity, low cost, and ability to replicate human biochemical and behavioral changes without causing physical harm [5, 53–57]. Furthermore, an attractive feature of restraint stress is that it is primarily a psychological stressor, i.e., movement restriction does not produce physical pain per se but involves anticipation of physical discomfort [58, 59]. Using this model, we examined stress-induced changes in maternal circulating sEVs and their potential impacts on placental/fetal tissues. Considering the potential role of maternal sEVS as stress-transferring signals to the placenta/fetus can pave the way for developing novel diagnostic tools and/or interventions to mitigate adverse outcomes in offspring.

## Materials and methods

### Animals

Nulliparous young adult female Sprague-Dawley rats (7–8 weeks old; 200–250 g body weight) were kept at 2 animals per cage. Once mating was confirmed, they were single-housed, as described in the stress protocol section. Rats were maintained at 22 °C, under a 12 h light/dark cycle, with ad libitum access to food (LabDiet 5P00 RMH3000) and water. As environmental enrichment, a cardboard cylinder, shredded paper, and nesting material were provided. In this study, 30 control rats and 44 rats subjected to the prenatal stress protocol were used. The experimental procedures were supervised and approved by the Scientific Ethics Committee of the Universidad de los Andes, Santiago, Chile (Folio #: CEC2021036), and performed following the National Institutes of Health Guide for the Care and Use of Laboratory Animals (8th Edition).

### Fetal sex determination

Fetal DNA was extracted following the HotSHOT protocol described by Truett et al. [60]. Sex determination PCR was performed following Dhakar et al. protocol [61].

### Stress protocol

The stress protocol used was previously described in males [46]. Pregnant rats subjected to control or restraint stress protocols were single-housed. Rats were habituated for 5 days (from gestational day GD0.5 to GD5.5). During habituation, animals were maintained ad libitum with freshly prepared 1% sucrose solution and water. At the end of GD5.5, the water and the sucrose solution were removed for 12–14 h. After this period, a sucrose preference test (SPT) was performed to determine the anhedonic state before the start of movement restriction. SPT consisted in measuring the preference rate of sucrose vs. water during 1 h-period. From stage GD6.5 to GD15.5, rats in the restraint stress group were confined to a wire box of 8 × 8 × 23 cm for a 2 h-period without access to food or water. In parallel, the control group was subjected to handling and withdrawing water and food during the same 2 h-period. At GD16.5, a second SPT was performed. Both groups were euthanized at GD18.5 by intraperitoneal injection of Ketamine 100 mg/kg body weight and xylazine 30 mg/Kg body weight and decapitation.

### Isolation of small EVs from maternal plasma

Peripheral blood was obtained by decapitation, collected in tubes (BD Vacutainer (ACD) solution A, 364606), and centrifuged at 2000 g for 30 min and 10,000 g for 45 min to obtain cell-free plasma. Pre-processed plasma was stored at -80 °C until extraction of sEVs. sEVs were obtained by ultracentrifugation following the protocol by Thery et al. [62]. In brief, 4-6mL of cell-free plasma were ultracentrifuged at 150,000 g x 2 h, washed with 10 mL of cold PBS 1X, and followed by a second ultracentrifugation at 150,000 g x 2 h. The resulting sEV-enriched fraction was resuspended in 100uL cold 1x PBS.

### Nanoparticle tracking analysis (NTA)

The size distribution and concentration of the sEVs were analyzed with a NanoSight NS-300 equipment (Malvern Instruments), using a green laser. sEVs were diluted in fresh prepared 0,22 μm filtrated 1x PBS in a range between 1:100 to 1:1000 (to obtain 20–100 particles per frame) to a final volume of 1mL. Five 1-min long videos were recorded per sample under the following condition: 25 °C module temperature, camera level 8, manual sample injection (NTA 3.2 Software). After capture, videos were analyzed by in-build Nanosight Software NTA 3.2 with a detection threshold of 3. An average of five videos was used for analysis.

### Western blot analysis

Small EVs samples were resuspended in 50mM HEPES, 0,15% SDS buffer, and protein quantification was measured using the Bicinchoninic acid method (BCA, Pierce^™^, Thermo Fisher Scientific, 23229), according to manufacturer instructions. Proteins were separated using sodium dodecyl sulfate-polyacrylamide gels (SDS-PAGE) under denaturing conditions and electroblotted onto Nitrocellulose Membrane (Thermo Fisher Scientific, 88018), which were blocked with 5% w/v skim milk in PBS tween 0,1% for 1 h at room temperature under constant agitation. Membranes were probed overnight at 4 °C with Flotilin-1 (1:500, 610821 BD Transduction Laboratories), CD63 (1:250, sc-95 15363, Santa Cruz Biotechnology); TSG101 (1:1000, 612697 BD Transduction Laboratories); GM130 (1:1000, 610823 BD Transduction Laboratories); GFAP (1:250, Mab C 2032-28B, US Biological); Aldolase C (1:250, sc-12065, Santa Cruz Biotechnology); EAAT2 (1:500, AGC-022, Alomone) in 1X PBS. Next, membranes were incubated for 1 h at room temperature with horseradish peroxidase (HRP)-conjugated secondary antibodies at 1:5,000. Finally, the blots were developed with the chemiluminescent reaction solution (Pierce^™^ ECL Western blotting Substrate) and visualized using the UVP ChemStudio PLUS equipment. Bands were quantified by densitometry using ImageJ software.

### DIR staining of sEVs

Plasma-derived sEVs from control or stressed pregnant rats (4 animals per condition) were pooled and stained by incubation at 37 °C for 1 h with 71 µM of DiR^®^ lipophilic marker (1,1-dioctadecyl-3,3,3,3-tetramethylindotricabocyanine iodide) (Invitrogen D1273), under 600 RPM shaking in dark conditions. Subsequently, DiR-labeled sEVs were ultracentrifuged at 120,000 g for 2 h, resuspended in sterile PBS pH = 7.4, and stored at -80 °C until later use. As a control, 71 µM DiR in PBS was used following the same procedure. After staining, the size distribution and concentration of DiR-sEVs were analyzed by NTA in a Nanosight-NS300 equipment. These DiR-sEVs were used as donor sEVs in biodistribution assays.

### Biodistribution assays using DiR-sEVs

Time-mating pregnant rats were divided into two recipient groups. Recipient control group was subjected to the control protocol mentioned above, and recipient stress group to the repetitive restraint stress protocol mentioned above. At gestational day 17.5, rats from both groups were intravenously injected under anesthesia (Ketamine 90 mg/kg and Xylazine 5 mg/kg) with 5 × 10^9^ particles/rat through the lateral tail vein. The scheme of injection was as follows: Control pregnant dams were injected with donor sEVs from control pregnant dams obtained at E17.5 (*n* = 4), and stressed pregnant dams were injected with donor sEVs from pregnant dams (E17.5) subjected to the stress protocol (*n* = 4). To further assess the role of sEVs and/or stressed tissues in biodistribution profile, crossed experiments were performed. For this, control pregnant dams were injected with donor sEVs from pregnant dams subjected to the stress protocol (*n* = 4), and stressed pregnant dams were injected with donor sEVs from control pregnant dams (*n* = 4). In all experimental groups, 24 h after the injection of DIR-labeled sEVs, the animals were anesthetized and perfused with 600 mL cold 1X PBS to remove blood. Mother’s organs (heart, lungs, liver, adrenal glands, and kidneys), fetuses, and placentas were dissected on ice and immediately transferred into cold 1X PBS in a dark container for image acquisition. Images were recorded using an Odyssey – CLx image acquisition equipment at a wavelength of 700 nm and 800 nm. The images were acquired and quantified using the Image StudioTM software (version 2.1) under the following parameters: image capture (automatic), depth (µ = 170 μm), quality (Q = high), focus (2.0 mm), length of wave (700 and 800 nm).

### Statistical analysis

Normality of the data was assessed using the D’Agostino–Pearson omnibus normality test. Mixed-effects modelling was used to compare between treatments while accounting for litter effects, with treatment as a fixed effect and litter as a random variable. Differences between two groups without mixed effects were determined using the Student’s t-test. Correlation between placental weight and fetal weight was assessed using Pearson’s correlation coefficient. The differences were considered statistically significant with a p-value < 0.05.

## Results

### A reliable model of maternal stress based on repetitive movement restriction

**Fig. 1:**
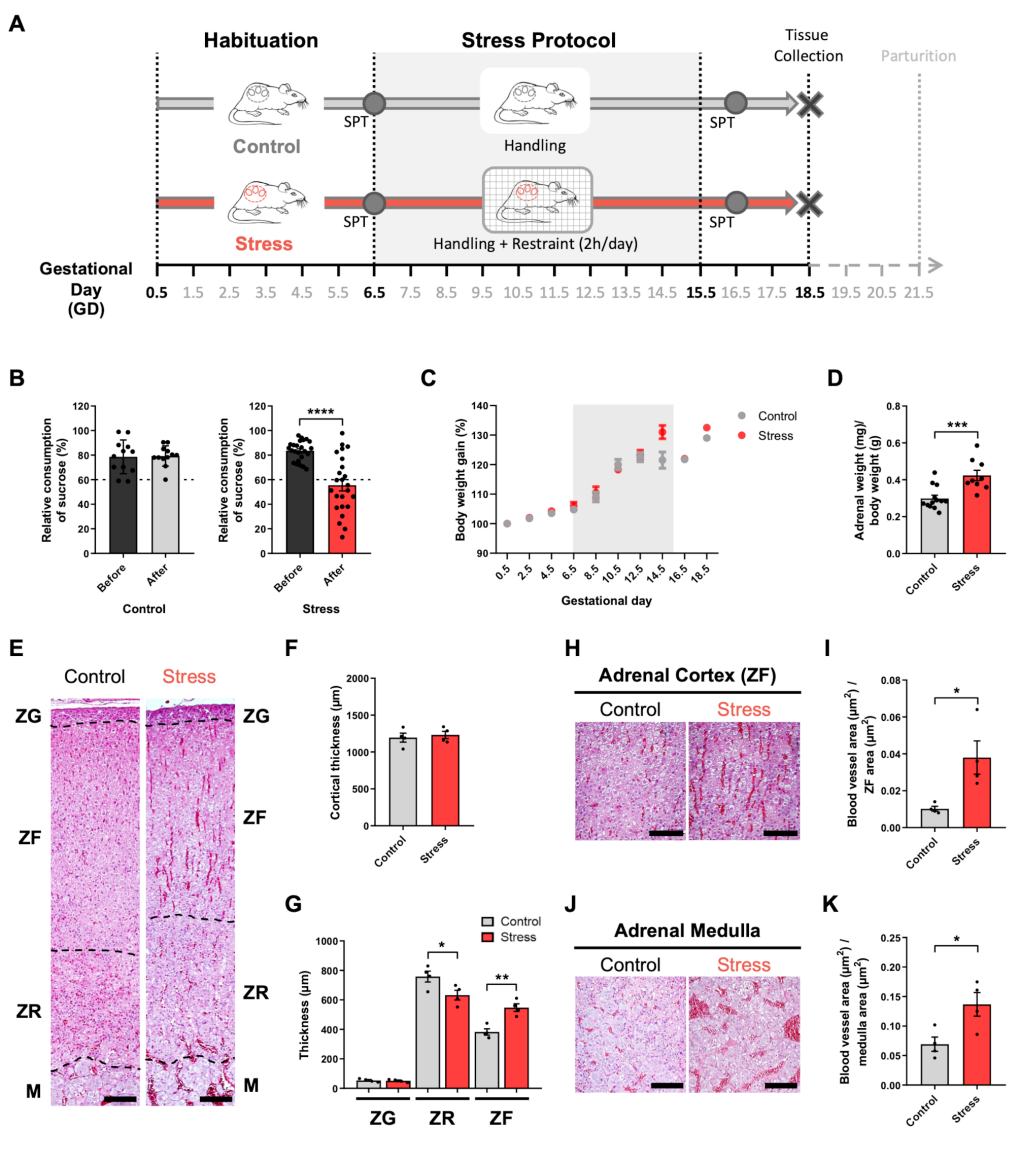
Repetitive restraint stress induces changes in maternal behavior and adrenal glands histology. **(A)** Schematic representation of the experimental restraint stress protocol. **(B)** Sucrose preference test (SPT) before (black bars) and after the restraint stress protocol (grey bar: control group; red bar: stress group). Relative sucrose versus water consumption is expressed as percentage (*n* = 12 control; *n* = 24 stress). **(C)** Maternal body weight gain expressed as percentage from gestational day (GD) 0.5. **(D)** Adrenal glands weight normalized by maternal body weight without uterus, placentas and fetuses/fetal membranes (*n* = 13 control, *n* = 9 stress). **(E-K)** Histological analysis of adrenal glands. **(E)** Hematoxylin-eosin staining of histological sections from adrenal glands. ZG: zona glomerulosa, ZF: zona fasciculata, ZR: zona reticularis, M: medulla. **(F-G)** Morphometric analysis of adrenal glands. Total cortical thickness **(F)** and thickness of different cortical layers **(G)** were measured in adrenal glands from control and stressed dams. **(H-K)** Representative images and quantification of the relative area of blood vessels in the adrenal cortex (zona fasciculata, ZF) (H-I) and in the adrenal medulla (J-K). Bars represent mean ± SEM (*n* = 4 control; *n* = 4 stress). * *p* < 0.05; ** *p* < 0.01; *** *p* < 0.001; **** *p* < 0.0001 (Student’s t-test). Scale bars: 100 μm

We used a repetitive restraint stress paradigm (2 h/day for 10 days, from GD6.5 to GD15.5) to model mild prenatal maternal stress during pregnancy, including placental organogenesis and initial fetal neurogenic period (Fig. 1A). Sucrose preference test (SPT) was performed before and after stress protocol to assess the level of anhedonia. As shown in Fig. 1B, stressed pregnant dams significantly decreased the preference for sucrose consumption compared with the non-stressed control females, indicating that our repetitive restraint stress protocol can induce stress-related behavior in pregnant dams. On the other hand, the evaluation of body weight gain showed no significant differences between stressed and unstressed animals (Fig. 1C), underscoring the mild intensity of the stress protocol used. Remarkably, adrenal glands weight at GD18.5 (adjusted by female weight excluding placentas and fetuses) showed significant differences between control and stress groups (Fig. 1D). In addition, histological analyses of adrenal glands showed: (i) no differences in cortical thickness (Fig. 1E-F); (ii) increased thickness of the zona reticularis with a concomitant reduction of the zona fasciculata thickness (Fig. 1E, G); and (iii) enlarged/congested cortical and medullary capillaries (Fig. 1H-I and J-K, respectively).

### Repetitive restraint stress is sufficient to produce sex-biased intrauterine growth restriction

**Fig. 2:**
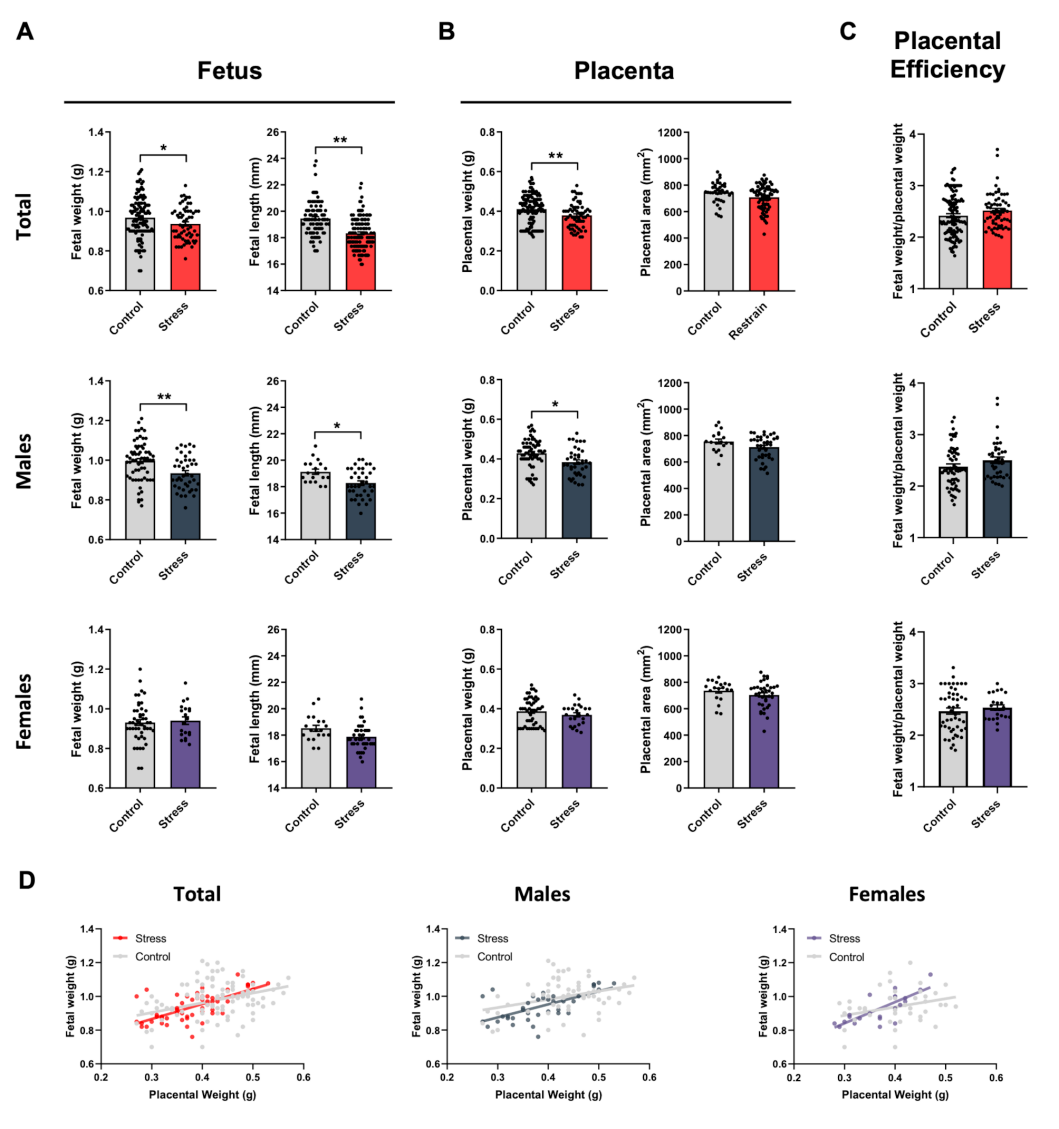
Repetitive restraint stress induces sex-biased effects in placental and fetal growth. **(A)** Fetal weight and length of total fetuses and separated by sex. **(B)** Placental weight and area of total placentas and separated by sex. **(C)** Placental efficiency. Bars represent mean ± SEM. In fetal/placental weight analyses: Control group: *n* = 114 fetuses/placentas (65 males and 49 females) from 8 different control dams; Stress group: *n* = 62 fetuses/placentas (42 males and 20 females) from 4 different stressed dams). In fetal length and placental area analyses: Control group: *n* = 38 fetuses/placentas (19 males and 19 females) from 4 different control dams; Stress group: 75 fetuses/placentas (38 males and 37 females) from 5 stressed dams). * *p* < 0.05; ** *p* < 0.01 (Statistical comparisons by mixed-effects modelling to control for litter effects; treatment (stress) was used as fixed effect and litter as random effect). **(D)** Correlation of fetal weight vs. placental weight in control (grey) and stress (red) groups. Total control group: y = 0.5783x + 0.73, *r* = 0.4045, r^2^ = 0.1636, *p* < 0.0001; Total Stress group: y = 0.8920x + 0.59; *r* = 0.6776, r^2^ = 0.4592, *p* < 0.0001; Males control group: y = 0.4874x + 0.7871, *r* = 0.3488, r^2^ = 0.1217, *p* = 0.0044; Male Stress group: y = 0.7761x + 0.6422, *r* = 0.6479, r^2^ = 0.4198, *p* = 0.0044; Female Control group: y = 0.4955x + 0.7388, *r* = 0.3430, r^2^ = 0.1177, *p* = 0.0158; Female Stress group: y = 1.258x + 0.4634, *r* = 0.7615, r^2^ = 0.58, *p* < 0.0001

We then evaluated whether repetitive maternal restraint stress induces changes in perinatal outcomes. No differences were observed in the number of embryos per litter, embryo viability, nor sex ratio of embryos between stressed pregnant and control pregnant dams (Suppl. Figure 1). We assessed fetal weight and length, and placental weight and area in fresh tissues at E18.5 controlling for fetal/placental sex. Fetal length and placental area were evaluated in images obtained under Odyssey–CLx imager. Interestingly, we found a reduction in fetal weight and length (Fig. 2A) and a reduction in placental weight in the group exposed to stress (Fig. 2B), suggesting that the repetitive stress protocol induces intrauterine growth restriction (IUGR). We further analyzed whether these changes were sex biased. Remarkably, the observed effect of maternal restraint stress on fetal and placental weight was greater in males than females. In fact, only male placentas/fetuses exposed to stress showed a significant weight reduction and no significant decrease was observed in fetal and placental weight of stress-exposed females (Fig. 2A-B). Similarly, a significant reduction of fetal length under stress conditions was observed only in males (Fig. 2A). Finally, no statistically significant differences were observed when placental areas were compared, controlling for sex (Fig. 2B) and no changes were observed in placental efficiency (fetal weight/placental weight) when comparing stressed and unstressed pregnant dams (Fig. 2C), nor when comparing this index in males and females, separately. On the other hand, linear regression modelling of fetal weight and placental weight showed a positive and significant correlation between both variables in the two groups of animals (Total control group: y = 0.5783x + 0.73, *r* = 0.4045, r^2^ = 0.1636, *p* < 0.0001; Total Stress group: y = 0.8920x + 0.59; *r* = 0.6776, r^2^ = 0.4592, *p* < 0.0001). However, the proportion of the variance for fetal weight (dependent variable) that is explained by placental weight (independent variable) was relatively low in control dams (r^2^ = 0.1636) and increased under stress conditions (r^2^ = 0.4592). Furthermore, when males and females were analyzed separately, we found that, under control conditions, the proportion of the variance for fetal weight that is explained by placental weight was similar in males and females (r^2^ = 0.1177 in female control group vs. r^2^ = 0.1217 in male control group). Remarkably, under stress conditions, the r^2^ value was higher in females (r^2^ = 0.58) than males (r^2^ = 0.4198) (Fig. 2D), suggesting that under stress conditions, there is a higher strength of the relationship between placental weight and fetal weight, and it is even higher in females than males.

### Repetitive restraint stress impacts maternal circulating sEVs

**Fig. 3:**
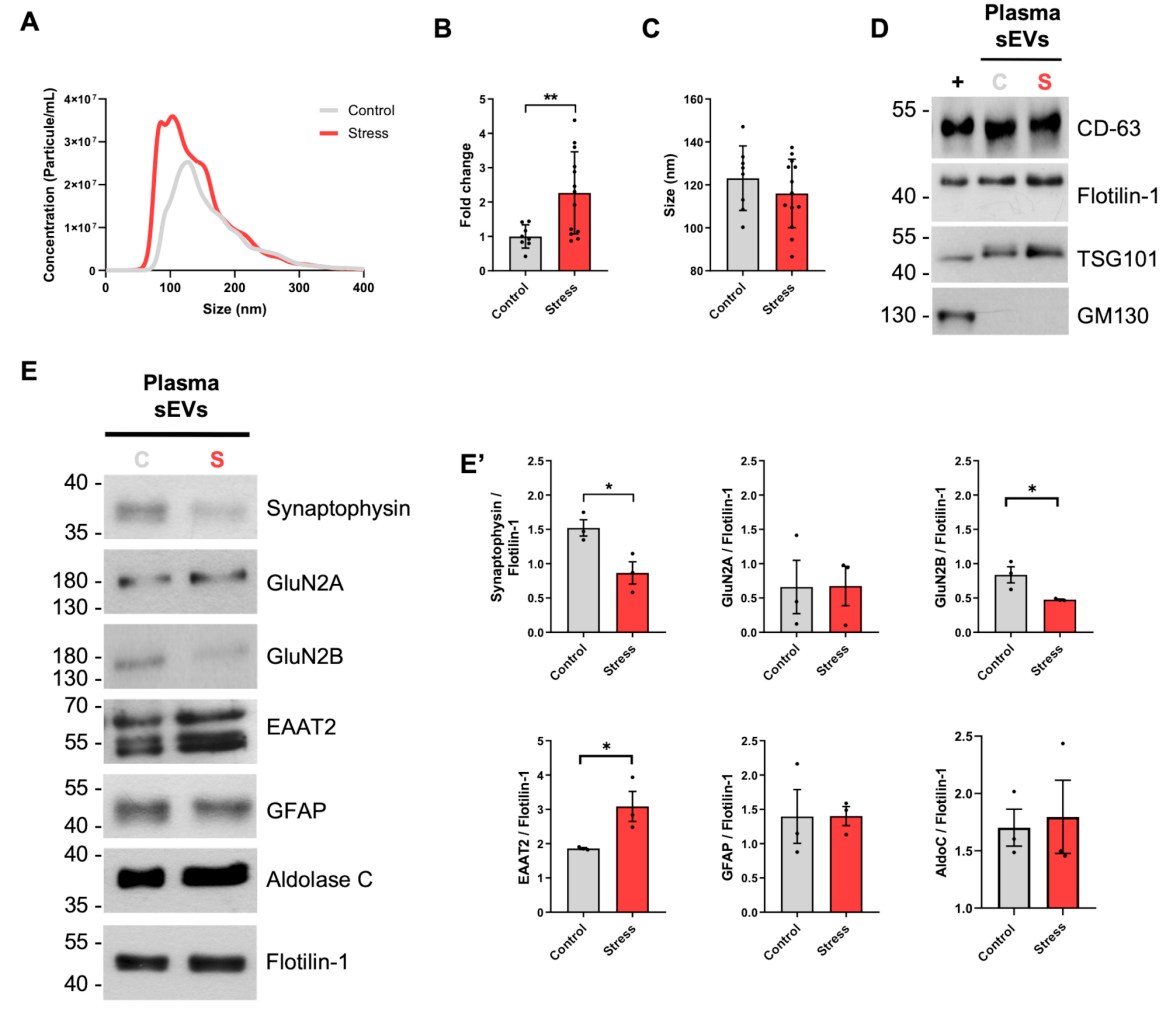
Repetitive restraint stress induces changes in concentration and cargo of maternal circulating sEVs. **(A)** Size and concentration distribution profile of circulating blood plasma-borne sEVs from control and stressed pregnant dams. **(B)** Concentration (particles/ml) of blood plasma-borne sEVs in control and stressed pregnant dams. **(C)** Size (mode) of blood plasma-borne sEVs in control and stressed pregnant dams. Bars represent mean ± SEM (Control group: n = 9; Stress group: n = 13). **(D)** Representative images of Western blot analyses for characterization of blood plasma-borne sEVs from control (C) and stressed (S) pregnant dams. Positive (CD-63, flotillin-1, TSG-101) and negative (GM130) markers for sEVS were used for characterization. Rat brain protein homogenates were used as positive controls (+). (E-E’) Western blot analyses of maternal blood plasma-borne sEVs for brain neuronal-enriched (synaptophysin, GluN2A, Glun2B) and astrocyte-enriched (EAAT2, GFAP, Aldolase C) proteins. Representative images of Western blots (E) and densitometric quantification analyses (E’) are shown. Bars represent mean ± SEM (Control and Stress groups: *n* = 3 pools of plasma-borne sEVs; each pool is composed by plasma-derived sEVS from 4 different pregnant rats). * *p* < 0.05; ** *p* < 0.01 (Student’s t-test)

We characterized circulating sEVs from maternal plasma of unstressed pregnant rats (controls) and pregnant rats subjected to the restraint stress protocol. Nanoparticle tracking analysis demonstrated an increment in plasma sEVs concentration (Fig. 3B) with no significant differences in the size mode (Fig. 3C) between control and stressed pregnant rats. Size distribution versus concentration plots suggest that the increased concentration of circulating sEVs is mainly due to smaller sEVs (sEVs of approximately 50–100 nm). We also characterized circulating plasma-derived sEVs from stressed and unstressed pregnant dams by Western blot using different markers known to be cargoes of sEVs, such as CD63, Flotilin-1, and TSG-101. TG130 was used as a negative control to exclude highly contaminated samples (Fig. 3D).

On the other hand, to test whether our restraint stress model in pregnant rats induces changes in the cargo of circulating sEVs, we decided to evaluate changes in the protein cargo of sEVs under stress. Since we wanted to test stress-related changes, we focused on evaluating brain-derived proteins previously described as genes/proteins associated with stress response [47, 63–68]. For this purpose, we decided to test proteins of neuronal origin: synaptophysin, GluN2A, GluN2B [63–67], and proteins of astroglial origin: EAAT2, GFAP, and Aldolase-C [47, 68]. Flotilin-1 was used as a loading control to normalize the levels of other protein cargoes. Interestingly, a significant reduction in neuron-enriched proteins (synaptophysin and GluN2B) and a significant increase in astrocyte-enriched proteins (EAAT2) were observed in blood-borne sEVs from stressed dams (Fig. 3E-E’).

### Biodistribution of plasma-derived sEVs in placental tissues is influenced by PS

Since we identified that repetitive restraint stress (i) increased the concentration and modified the cargo of maternal circulating sEVs, and (ii) induced a fetal/placental growth reduction, we decided to investigate whether maternal circulating sEVs can target placental and/or fetal tissues and whether this biodistribution (mother-to-fetus communication) is affected by repetitive restraint stress.

**Fig. 4:**
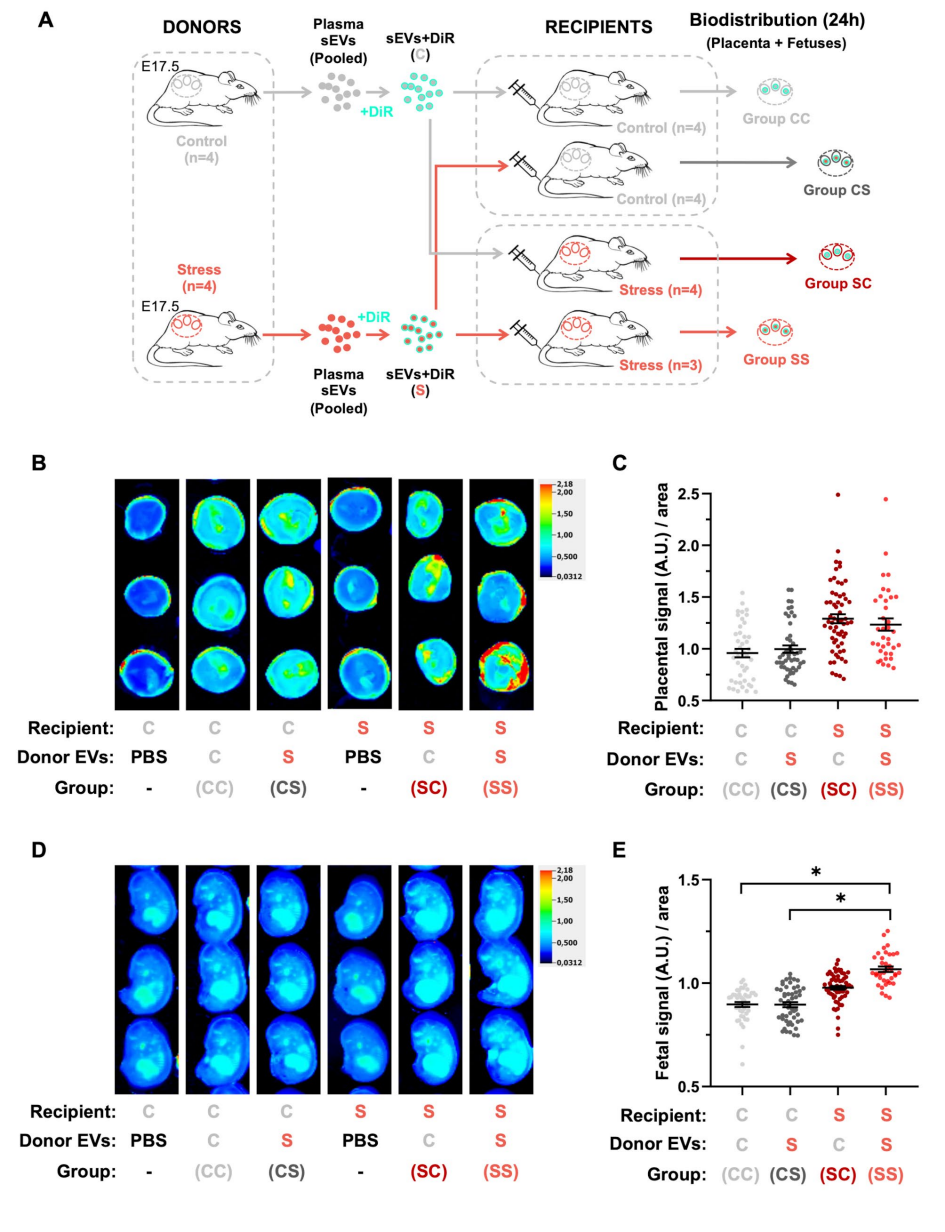
Repetitive restraint stress affect biodistribution of maternal circulating sEVS into placental and fetal tissues. **(A)** Schematic representation of the experimental design. Plasma-borne sEVs from 4 control rats or 4 stressed rats at GD17.5 were isolated, pooled, and stained with the lipophilic marker DiR. Labelled-sEVS from both stressed and control rats (Donors) were intravenously (tail vein) injected into pregnant stressed and pregnant control recipient rats at E17.5 and analyzed after 24 h (at 18.5). **(B**,** D)** Representative images of DiR fluorescent signal distribution in placentas (B) and fetuses (D) 24 h after labelled-sEVs injection. PBS-DiR was used as negative control. **(C**,** E)** Quantification of DiR fluorescent signals in placentas (C) and fetuses (E) from Group CC (control recipients that received control donor sEVs), Group SS (stressed recipients that received donor sEVs from stressed pregnant dams), Group CS (control recipients that received donor sEvs from stressed dams), and Group SC (stressed recipients that received control donor sEVs). Fluorescence intensity (arbitrary units, A.U.) was normalized by placental and fetal area, respectively. Data shown as scatter dot plots with mean ± SEM. *n* = 44 placentas/fetuses from 4 litters for Group CC; *n* = 36 placentas/fetuses from 3 litters for Group SS; *n* = 50 placentas/fetuses from 4 litters for Group CS; *n* = 62 placentas/fetuses from 4 litters for Group SC. * *p* < 0.05 (Statistical comparisons by mixed-effects modelling to control for litter effects; treatments were used as fixed effect and litter as random effect)

Plasma-derived sEVs from pregnant dams previously exposed to the restraint stress or control protocol (*n* = 4 in each group) were obtained at E17.5, pooled, and stained with DiR (Donor sEVs). To assess biodistribution, each DIR-stained pool of sEVs was characterized by NTA and injected intravenously (tail vein) into E17.5 pregnant females previously exposed to the control or restraint stress protocol (Recipients; *n* = 4 in each group) (Fig. 4A). Biodistribution of DiR signals was assessed in maternal organs as a technical control. Interestingly, no differences were observed in the biodistribution profile of DiR-sEVs in maternal organs, and, in all experimental groups, the liver was the organ with the higher relative accumulation of DiR + signals (Suppl. Figure 2 A-B).

Remarkably, when biodistribution was assessed in placentas (Fig. 4B-C) and fetuses (Fig. 4D-E), we found that placentas and fetuses from stressed dams injected with sEVs from stressed dams (SS) showed increased DiR + signals compared with control dams injected with control sEVs (CC); however, these differences were statistically significant only in fetuses (Fig. 4C, E; compare Group CC vs. Group SS). These results suggest that under stress conditions, maternal circulating sEVs can distribute more efficiently and/or accumulate in placental/fetal tissues than under control conditions. To investigate whether this phenomenon is due to stress-induced changes in circulating sEVs (making them target placental/fetal tissues more efficiently) and/or stress-induced changes in placental/fetal tissues (making them more receptive to circulating sEVs), we performed crossed experiments as shown in Fig. 4A (i.e., control dams injected with donor sEVs from stressed pregnant rats (Group CS) and stressed dams injected with sEVs from control pregnant animals (Group SC). The results showed a trend towards a higher distribution/accumulation of control sEVs in stressed placentas and fetuses (Group CS) than control placentas/fetuses (Group CC); however, no statistical significance was observed (Fig. 4C, E). Interestingly, no changes were observed in the biodistribution of sEVs from stressed dams in control placentas/fetuses (Group CS) when compared with the biodistribution of sEVs from control dams in control placentasl/fetuses (Group CC) (Fig. 4C, E). On the other hand, when sEVs from stressed pregnant dams were injected in control animals (Group CS), we found that they distribute/accumulate less in placentas/fetuses from control dams than in placentas/fetuses from stressed dams (Group SS), being statistically significant only in fetuses (Fig. 4C, E). No statistical differences were observed in biodistribution of sEVs from stressed dams and sEVs from control dams when they were injected in stressed dams (Fig. 4C, E; compare Group SS with Group SC). Remarkably, in all the experiments, DiR + signals were more intense in placentas than in fetuses (Suppl. Figure 2 C-D).

## Discussion

The crosstalk between the mother and the fetus across the placenta plays a critical role in the success of pregnancy and the developmental outcome of the offspring. Here, we report that maternal circulating sEVs change their concentration and cargo after repetitive restraint stress exposure. We also demonstrate that maternal circulating sEVs target placental and fetal tissues. Furthermore, placentas and fetuses from stressed dams appear more receptive to maternal circulating sEVs. Together, these results suggest that maternal sEVs could mediate and/or modulate stress signals from the mother to the fetus, thus acting as relevant actors in stress-mediated fetal programming.

As stated previously, among the different types of stressors used in rodents [55, 56], the restraint stress protocol is a preferred method of stressing rodent pregnant dams [54, 56, 57] and is particularly appealing because (i) even though it involves a physical component, it is primarily a psychological stressor [58, 59], and (ii) the offspring of rodents subjected to paradigms that involve restraint stress during pregnancy show a higher risk for developing adverse neurodevelopmental outcomes in postnatal life, such as anxiety-like behavior [69–72], depressive-like symptoms [70, 72–74], and cognitive impairment [75–77]. In our model, pregnant dams subjected to repetitive restraint stress showed increased anhedonic behavior and increased adrenal weight, along with in our model, pregnant dams subjected to repetitive restraint stress showed increased anhedonic behavior and increased adrenal weight, along with increased relative thickness of the cortical zona reticularis, and congestive cortical and medullary vessels of these glands. Remarkably, those histological changes have been previously described in the adrenal glands of rats chronically exposed to different stressors [78–82]; thus, suggesting that our protocol is effective. On the other hand, body weight gain was not significantly different across groups after stress protocol, suggesting that our stress protocol is mild and does not affect food intake in pregnant dams [83].

Several studies have demonstrated that prenatal stress and/or increased maternal glucocorticoids negatively affect fetal and/or placental growth [84, 85]. Furthermore, these responses appear to be dependent not only on the stressor’s severity but on exposure timing (gestational age) and fetal sex [25, 32, 86–89]. In our model, male fetuses and placentas were more significantly affected than females. These results agree with other studies, suggesting that, in our stress-exposure timing, male fetuses/placentas are less adaptable or more susceptible to maternal stress signals than female fetuses/placentas [34, 36]. In this context, even though maternal stress “signals” such as glucocorticoids can potentially cross the placenta and directly impact developing fetal tissues, placental cells appear to be involved in mediating or communicating maternal milieu changes to fetal developing tissues [90–93]. The placenta resides at the maternal-fetal interface, so it is uniquely positioned to mediate interactions within an adverse intrauterine environment. Impairment of placental organogenesis [94] or disruption of its critical functions can broadly impact fetal development, conferring lasting effects on developing organs [6, 95]. Placental function is regulated by the collective response of its cells to the local environment [96]; thus, a disruption of the maternal milieu by stress or other stimuli can adversely influence placental structure and function. Furthermore, as the placenta is formed by cells (trophoblasts) derived from the fetus, it expresses the fetal genetic sex [97], which determines sex-dependent differences in size and gene expression [91, 98–101]. Such basal placental sex differences likely enable sex-specific responses to normal and pathologic environments. In this context, exposing pregnant mice to stress at early gestational stages induces a pro-inflammatory response within the placenta, that mainly affects males [102]. Different levels of evidence are in line with these findings. For instance, the expression of several genes that regulate placental function, nutrient transport, and glucocorticoid metabolism, is more severely affected in males than females placentas after PS [93, 103–105]. Also, PS can produce sex-specific changes (male placentas more affected than females) that are also observed in placentas from aged rats [106], including a reduced expression of growth-regulatory genes [104], increased placental oxidative stress [106, 107], increased mRNA levels of the pro-apoptotic genes, and specific histopathological changes [102, 106, 108]. Together, all these data support that the placenta is a crucial target of maternal stress and can mediate, at least in part, lasting sex-specific consequences in offspring development.

The mechanisms by which PS induces functional and/or structural changes in the placenta and/or the fetus remain poorly understood. Small EVs are now recognized as potent mediators of intercellular communication, capable of transferring various cellular components from donor to recipient cells through paracrine and endocrine pathways [44]. Several articles suggest that maternal circulating sEVs increase in number [48, 49] and change their composition with pregnancy (i.e., comparing sEV cargo of pregnant versus non-pregnant women) [109] and with pregnancy progression (i.e., with gestational age) [49, 110]; thus, suggesting a relevant role of maternal circulating sEVs in the physiology of pregnancy. Furthermore, maternal circulating sEVs may also reflect pregnancy-related disorders and be used as biomarkers [111, 112]. These data, emphasizes that sEVs could act as a potential bidirectional maternal-fetal communication mechanism. In this sense, it has been proposed that a significant fraction of the changes in maternal circulating sEVs during pregnancy are originated by placenta-derived sEVs [49, 113]. In fact, a growing body of evidence suggests that placental sEVs can modulate the maternal immune system during pregnancy [114]. On the other hand, maternal-derived sEVs can communicate signals from maternal tissues to the placenta and/or the fetus. Consistent with this, here we showed that maternal circulating sEVs target placental and fetal tissues, thus potentially acting as mediators or modulators of mother-to-fetus communication. Interestingly, we also showed that maternal stress during pregnancy increased the arrival and/or retention of maternal sEVs in the placenta and the fetus, suggesting that under chronic stress conditions, these tissues are more receptive to maternal circulating sEVs. As stated before, several studies in experimental animals have addressed molecular, histological, and functional changes in the placenta of stressed pregnant dams [102, 106, 108]. These changes could explain an increased receptiveness of placental and fetal tissues to maternal sEVs; however, further studies should be performed to determine the precise relationship between maternal sEVs and placental/fetal changes under stress conditions.

In addition to the increased distribution of maternal sEVs in stressed placental/fetal tissues, we also observed that repetitive restrain stress increased the concentration of circulating sEVs, notably smaller sEVs. Together, these changes could reflect modifications in the release and/or uptake of sEVs by maternal and placental/fetal tissues under stress conditions; and highlight the role of circulating sEVs as potential stress signals. Small EVs carry a set of proteins, RNAs, and lipids unique to the cells that secrete them and unique to the physiological or pathological context of these cells [115–117]. In this context, several authors have proposed that sEVs can be relevant in the pathophysiology of several disorders and valuable as biomarkers for the diagnosis and/or prognosis of those disorders [118, 119]. Consistent with this, our research group have previously showed that circulating sEVs in the peripheral blood carried a particular protein cargo composition that reflected the exposure to different stress conditions [47].

In the present study, we demonstrate that maternal circulating sEVs include brain astrocyte-derived sEVs (ADEVs) that contain a specific cargo of astrocyte-enriched proteins, such as the glycolytic enzyme Aldolase C, GFAP, and the astroglial glutamate transporter EAAT2 [47, 120, 121], and neuron-derived sEVs (NDEVs) that contain, among others, synaptic proteins (synaptophysin, glutamate receptor subunits). Remarkably, under restraint stress conditions, we found increased levels of astrocyte-enriched proteins such as EAAT2, compared to control pregnant dams. These results are in agreement with previous studies in adult male rats, which described that astrocyte-enriched proteins were increased in circulating sEVs after restraint stress [47] and suggest that, under chronic repetitive stress conditions, ADEVs are either increased in number or contain a higher cargo of proteins selectively expressed in astrocytes.

On the other hand, it is well-documented that chronic stress modifies neuronal (synaptic) activity in several brain regions, leading to stress-induced changes that generate local and systemic outputs [122, 123]. For instance, chronic stress or dexamethasone exposure decreases the protein expression of the post-synaptic NMDA-type ionotropic glutamate receptor (NMDAR) subunit GluN2B in the hippocampus of mice [63, 64]. Similarly, Yuen et al. observed that five consecutive days of stress exposure were enough to decrease the total and surface amount of GluN1, GluN2A, and GluN2B subunits in the prefrontal cortex of stressed brains via a glucocorticoid receptor-dependent process [65]. On the other hand, several studies have demonstrated that chronic (repetitive) restraint stress leads to reduced synaptophysin expression (mRNA and protein) in the hippocampus of stressed rats [66, 67]. In the present study, we observed that circulating sEVs from pregnant stressed dams showed decreased levels of neuron(synapsis)-enriched proteins such as synaptophysin and GluN2B. To our knowledge, the present study is the first to demonstrate such changes in the cargo of circulating sEVs in response to maternal stress during pregnancy. Furthermore, these results strongly suggest that changes in the protein cargo composition of circulating sEVs reflect stress-induced biochemical changes in the maternal brain and, consequently, could act as “stress signals” able to target peripheral tissues. This phenomenon has also been proposed by a study showing that repetitive restraint stress provoked a downregulation of miR-26a in brain hippocampal tissue and blood-borne sEVs concomitantly [124].

Finally, in line with our findings, recent evidence suggests that maternal-fetal communication may be mediated in part by sEVs. In this context, Sheller-Miller et al. demonstrated in a clever experiment using a Cre-reporter mouse model that placental cells can uptake maternal sEVs. In addition, they also demonstrate that maternal sEVs can cross the placenta, traffic into fetal tissues, and induce functional changes in these tissues [50]. On the other hand, using a strategy similar to the one used in our study, two independent studies isolated sEVs from maternal sources (blood and adipose tissue) of pregnant mice, labeled them with fluorescent dyes, and injected them back intravenously (tail vein) into pregnant mice [51, 52]. Remarkably, they found fluorescent signals in the placenta and fetal tissues, suggesting that circulating maternal sEVs target the placenta and cross the placental barrier to be delivered into fetal tissues [51, 52]. In this context, our results advance the understanding of maternal-fetal communication, adding novel and relevant data to stress-dependent changes in circulating sEVs and their targeting to placental/fetal tissues, and highlighting the role of maternal sEVs as signals that can mediate, modulate and/or potentiate stress-induced changes in those tissues.

One limitation of this study is that it is conducted in a rodent model, which may not fully replicate the complexities of human pregnancy and stress responses. On the other hand, while it identifies PS-induced changes in sEV concentration, composition, and biodistribution, it does not fully elucidate the specific molecular mechanisms by which these vesicles influence placental and fetal tissues, nor the cumulative effect of sEVs at different gestational stages.

Future research is needed to identify the cellular targets of maternal circulating sEVs in placental/fetal tissues, as well as the specific cargo within maternal sEVs that mediates their effects under stress conditions. Focusing on stress-induced changes in ADEVs within this framework could serve as a good starting point. Additionally, expanding this research to human studies will be crucial for translating these findings into clinical applications. Future studies should explore the potential for using sEVs as biomarkers for early detection of stress-related pregnancy complications. Also, investigating therapeutic interventions that can modulate sEV release or function may offer new avenues for preventing PS-related adverse developmental outcomes.

## Electronic supplementary material

Below is the link to the electronic supplementary material.

Supplementary Material 1

Supplementary Material 2

## Data Availability

The datasets used and/or analysed during the current study are available from the corresponding author on reasonable request.

## Abbreviations

sEVs: Small extracellular vesicles
PS: Prenatal stress
GD: Gestational day
SPT: Sucrose preference test
NTA: Nanoparticle tracking analysis
IUGR: Intrauterine growth restriction
ADEVs: Astrocyte-derived small extracellular vesicles
NDEVs: Neuron-derived sEVs
TSG101: Tumor Susceptibility 101
GM130: cis-Golgi Marker
GFAP: Glial Fibrillary Acidic Protein
EAAT2: Solute carrier family 1 member 2
Glun2A: Glutamate ionotropic receptor NMDA type subunit 2 A
Glun2B: Glutamate ionotropic receptor NMDA type subunit 2B
AldoC: Fructose-Bisphosphate Aldolase C

## References

[1] Barker DJ. The fetal and infant origins of adult disease. BMJ. 1990;301:1111. 2252919 10.1136/bmj.301.6761.1111 PMC1664286

[2] Bock J, Rether K, Groger N, Xie L, Braun K. Perinatal programming of emotional brain circuits: an integrative view from systems to molecules. Front Neurosci. 2014;8:11. 24550772 10.3389/fnins.2014.00011 PMC3913903

[3] Bock J, Wainstock T, Braun K, Segal M. Stress in Utero: prenatal programming of Brain plasticity and cognition. Biol Psychiatry. 2015;78:315–26. 25863359 10.1016/j.biopsych.2015.02.036

[4] Markham JA, Koenig JI. Prenatal stress: role in psychotic and depressive diseases. Psychopharmacology. 2011;214:89–106. 20949351 10.1007/s00213-010-2035-0 PMC3050113

[5] Weinstock M. The long-term behavioural consequences of prenatal stress. Neurosci Biobehav Rev. 2008;32:1073–86. 18423592 10.1016/j.neubiorev.2008.03.002

[6] Bronson SL, Bale TL. The Placenta as a mediator of stress effects on Neurodevelopmental Reprogramming. Neuropsychopharmacology. 2016;41:207–18. 26250599 10.1038/npp.2015.231 PMC4677129

[7] Sandman CA, Davis EP, Buss C, Glynn LM. Exposure to prenatal psychobiological stress exerts programming influences on the mother and her fetus. Neuroendocrinology. 2012;95:7–21. 21494029 10.1159/000327017 PMC7068789

[8] Woody CA, Ferrari AJ, Siskind DJ, Whiteford HA, Harris MG. A systematic review and meta-regression of the prevalence and incidence of perinatal depression. J Affect Disord. 2017;219:86–92. 28531848 10.1016/j.jad.2017.05.003

[9] Dennis CL, Falah-Hassani K, Shiri R. Prevalence of antenatal and postnatal anxiety: systematic review and meta-analysis. Br J Psychiatry. 2017;210:315–23. 28302701 10.1192/bjp.bp.116.187179

[10] Lu YC, Andescavage N, Wu Y, Kapse K, Andersen NR, Quistorff J, et al. Maternal psychological distress during the COVID-19 pandemic and structural changes of the human fetal brain. Commun Med (Lond). 2022;2:47. 35647608 10.1038/s43856-022-00111-w PMC9135751

[11] Wu Y, Kapse K, Jacobs M, Niforatos-Andescavage N, Donofrio MT, Krishnan A et al. Association of maternal psychological distress with in Utero Brain Development in fetuses with congenital heart disease. JAMA Pediatr. 2020:e195316. 10.1001/jamapediatrics.2019.5316 PMC6990726 31930365

[12] Wu Y, Lu YC, Jacobs M, Pradhan S, Kapse K, Zhao L, et al. Association of prenatal maternal psychological distress with fetal brain growth, metabolism, and cortical maturation. JAMA Netw Open. 2020;3:e1919940. 31995213 10.1001/jamanetworkopen.2019.19940 PMC6991285

[13] Palmeiro-Silva YK, Orellana P, Venegas P, Monteiro L, Varas-Godoy M, Norwitz E, et al. Effects of earthquake on perinatal outcomes: a Chilean register-based study. PLoS ONE. 2018;13:e0191340. 29474413 10.1371/journal.pone.0191340 PMC5825031

[14] Paarlberg KM, Vingerhoets AJ, Passchier J, Dekker GA, Van Geijn HP. Psychosocial factors and pregnancy outcome: a review with emphasis on methodological issues. J Psychosom Res. 1995;39:563–95. 7490693 10.1016/0022-3999(95)00018-6

[15] Wainstock T, Lerner-Geva L, Glasser S, Shoham-Vardi I, Anteby EY. Prenatal stress and risk of spontaneous abortion. Psychosom Med. 2013;75:228–35. 23362503 10.1097/PSY.0b013e318280f5f3

[16] Kurki T, Hiilesmaa V, Raitasalo R, Mattila H, Ylikorkala O. Depression and anxiety in early pregnancy and risk for preeclampsia. Obstet Gynecol. 2000;95:487–90. 10725477 10.1016/s0029-7844(99)00602-x

[17] Ramchandani PG, Richter LM, Norris SA, Stein A. Maternal prenatal stress and later child behavioral problems in an urban South African setting. J Am Acad Child Adolesc Psychiatry. 2010;49:239–47. 20410713

[18] Talge NM, Neal C, Glover V, Early Stress TR, Prevention Science Network F et al. Neonatal Experience on C,. Antenatal maternal stress and long-term effects on child neurodevelopment: how and why? J Child Psychol Psychiatry. 2007;48:245 – 61. 10.1111/j.1469-7610.2006.01714.x PMC11016282 17355398

[19] Bergman K, Sarkar P, O’Connor TG, Modi N, Glover V. Maternal stress during pregnancy predicts cognitive ability and fearfulness in infancy. J Am Acad Child Adolesc Psychiatry. 2007;46:1454–63. 18049295 10.1097/chi.0b013e31814a62f6

[20] Davis EP, Sandman CA. The timing of prenatal exposure to maternal cortisol and psychosocial stress is associated with human infant cognitive development. Child Dev. 2010;81:131–48. 20331658 10.1111/j.1467-8624.2009.01385.x PMC2846100

[21] Torche F. Prenatal exposure to an Acute Stressor and Children’s cognitive outcomes. Demography. 2018;55:1611–39. 30105648 10.1007/s13524-018-0700-9

[22] Aizer A, Stroud L, Buka S. Maternal stress and child outcomes: evidence from siblings. J Hum Resour. 2016;51:523–55. 29118458 10.3386/w18422 PMC5673140

[23] Laplante DP, Brunet A, Schmitz N, Ciampi A, King S. Project Ice Storm: prenatal maternal stress affects cognitive and linguistic functioning in 5 1/2-year-old children. J Am Acad Child Adolesc Psychiatry. 2008;47:1063–72. 18665002 10.1097/CHI.0b013e31817eec80

[24] King S, Laplante DP. The effects of prenatal maternal stress on children’s cognitive development: Project Ice Storm. Stress. 2005;8:35–45. 16019596 10.1080/10253890500108391

[25] Batiz LF, Palmeiro-Silva YK, Rice GE, Monteiro LJ, Galaburda AM, Romero R, et al. Maternal exposure to a high-magnitude earthquake during pregnancy influences pre-reading skills in early childhood. Sci Rep. 2021;11:9244. 33927303 10.1038/s41598-021-88767-7 PMC8084950

[26] Guo C, He P, Song X, Zheng X. Long-term effects of prenatal exposure to earthquake on adult schizophrenia. Br J Psychiatry. 2019;215:730–5. 31113505 10.1192/bjp.2019.114 PMC7557601

[27] Malaspina D, Corcoran C, Kleinhaus KR, Perrin MC, Fennig S, Nahon D, et al. Acute maternal stress in pregnancy and schizophrenia in offspring: a cohort prospective study. BMC Psychiatry. 2008;8:71. 18717990 10.1186/1471-244X-8-71 PMC2546388

[28] Ronald A, Pennell CE, Whitehouse AJ. Prenatal Maternal Stress Associated with ADHD and autistic traits in early Childhood. Front Psychol. 2010;1:223. 21833278 10.3389/fpsyg.2010.00223 PMC3153828

[29] Buss C, Davis EP, Muftuler LT, Head K, Sandman CA. High pregnancy anxiety during mid-gestation is associated with decreased gray matter density in 6-9-year-old children. Psychoneuroendocrinology. 2010;35:141–53. 19674845 10.1016/j.psyneuen.2009.07.010 PMC2795128

[30] Buss C, Davis EP, Shahbaba B, Pruessner JC, Head K, Sandman CA. Maternal cortisol over the course of pregnancy and subsequent child amygdala and hippocampus volumes and affective problems. Proc Natl Acad Sci U S A. 2012;109:E1312–9. 22529357 10.1073/pnas.1201295109 PMC3356611

[31] Wen DJ, Poh JS, Ni SN, Chong YS, Chen H, Kwek K, et al. Influences of prenatal and postnatal maternal depression on amygdala volume and microstructure in young children. Transl Psychiatry. 2017;7:e1103. 28440816 10.1038/tp.2017.74 PMC5416711

[32] Bale TL. Sex differences in prenatal epigenetic programming of stress pathways. Stress. 2011;14:348–56. 21663536 10.3109/10253890.2011.586447

[33] Zagron G, Weinstock M. Maternal adrenal hormone secretion mediates behavioural alterations induced by prenatal stress in male and female rats. Behav Brain Res. 2006;175:323–8. 17023059 10.1016/j.bbr.2006.09.003

[34] Iturra-Mena AM, Arriagada-Solimano M, Luttecke-Anders A, Dagnino-Subiabre A. Effects of prenatal stress on anxiety- and depressive-like behaviours are sex-specific in prepubertal rats. J Neuroendocrinol. 2018;30:e12609. 29772083 10.1111/jne.12609

[35] Barbie-Shoshani Y, Shoham S, Bejar C, Weinstock M. Sex-specific effects of prenatal stress on memory and markers of neuronal activity in juvenile rats. Dev Neurosci. 2016;38:206–19. 27372837 10.1159/000446981

[36] Zuena AR, Mairesse J, Casolini P, Cinque C, Alema GS, Morley-Fletcher S, et al. Prenatal restraint stress generates two distinct behavioral and neurochemical profiles in male and female rats. PLoS ONE. 2008;3:e2170. 18478112 10.1371/journal.pone.0002170 PMC2366064

[37] Richardson HN, Zorrilla EP, Mandyam CD, Rivier CL. Exposure to repetitive versus varied stress during prenatal development generates two distinct anxiogenic and neuroendocrine profiles in adulthood. Endocrinology. 2006;147:2506–17. 16455779 10.1210/en.2005-1054

[38] Kaiser S, Sachser N. The effects of prenatal social stress on behaviour: mechanisms and function. Neurosci Biobehav Rev. 2005;29:283–94. 15811499 10.1016/j.neubiorev.2004.09.015

[39] Davis EP, Pfaff D. Sexually dimorphic responses to early adversity: implications for affective problems and autism spectrum disorder. Psychoneuroendocrinology. 2014;49:11–25. 25038479 10.1016/j.psyneuen.2014.06.014 PMC4165713

[40] Sandman CA, Glynn LM, Davis EP. Is there a viability-vulnerability tradeoff? Sex differences in fetal programming. J Psychosom Res. 2013;75:327–35. 24119938 10.1016/j.jpsychores.2013.07.009 PMC3796732

[41] Rakers F, Rupprecht S, Dreiling M, Bergmeier C, Witte OW, Schwab M. Transfer of maternal psychosocial stress to the fetus. Neurosci Biobehav Rev. 2017. 10.1016/j.neubiorev.2017.02.019 28237726

[42] Kalluri R, LeBleu VS. The biology, function, and biomedical applications of exosomes. Science. 2020;367. 10.1126/science.aau6977 PMC7717626 32029601

[43] Yanez-Mo M, Siljander PR, Andreu Z, Zavec AB, Borras FE, Buzas EI, et al. Biological properties of extracellular vesicles and their physiological functions. J Extracell Vesicles. 2015;4:27066. 25979354 10.3402/jev.v4.27066 PMC4433489

[44] Stahl PD, Raposo G. Extracellular vesicles: exosomes and Microvesicles, Integrators of Homeostasis. Physiol (Bethesda). 2019;34:169–77. 10.1152/physiol.00045.2018 30968753

[45] Kakarla R, Hur J, Kim YJ, Kim J, Chwae YJ. Apoptotic cell-derived exosomes: messages from dying cells. Exp Mol Med. 2020;52:1–6. 31915368 10.1038/s12276-019-0362-8 PMC7000698

[46] Ampuero E, Luarte A, Santibanez M, Varas-Godoy M, Toledo J, Diaz-Veliz G, et al. Two chronic stress models based on Movement restriction in rats respond selectively to antidepressant drugs: aldolase C as a potential biomarker. Int J Neuropsychopharmacol. 2015;18:pyv038. 25813018 10.1093/ijnp/pyv038 PMC4648154

[47] Gomez-Molina C, Sandoval M, Henzi R, Ramirez JP, Varas-Godoy M, Luarte A, et al. Small extracellular vesicles in rat serum Contain astrocyte-derived protein biomarkers of repetitive stress. Int J Neuropsychopharmacol. 2019;22:232–46. 30535257 10.1093/ijnp/pyy098 PMC6403096

[48] Mohammad S, Hutchinson KA, da Silva DF, Bhattacharjee J, McInnis K, Burger D, et al. Circulating small extracellular vesicles increase after an acute bout of moderate-intensity exercise in pregnant compared to non-pregnant women. Sci Rep. 2021;11:12615. 34135428 10.1038/s41598-021-92180-5 PMC8209031

[49] Salomon C, Torres MJ, Kobayashi M, Scholz-Romero K, Sobrevia L, Dobierzewska A, et al. A gestational profile of placental exosomes in maternal plasma and their effects on endothelial cell migration. PLoS ONE. 2014;9:e98667. 24905832 10.1371/journal.pone.0098667 PMC4048215

[50] Sheller-Miller S, Choi K, Choi C, Menon R. Cyclic-recombinase-reporter mouse model to determine exosome communication and function during pregnancy. Am J Obstet Gynecol. 2019;221:502e1. e12. 10.1016/j.ajog.2019.06.010 31207235

[51] Shi R, Zhao L, Cai W, Wei M, Zhou X, Yang G, et al. Maternal exosomes in diabetes contribute to the cardiac development deficiency. Biochem Biophys Res Commun. 2017;483:602–8. 27998767 10.1016/j.bbrc.2016.12.097

[52] Liu Y, Wang Y, Wang C, Shi R, Zhou X, Li Z, et al. Maternal obesity increases the risk of fetal cardiac dysfunction via visceral adipose tissue derived exosomes. Placenta. 2021;105:85–93. 33556718 10.1016/j.placenta.2021.01.020

[53] Fumagalli F, Molteni R, Racagni G, Riva MA. Stress during development: impact on neuroplasticity and relevance to psychopathology. Prog Neurobiol. 2007;81:197–217. 17350153 10.1016/j.pneurobio.2007.01.002

[54] Pallares ME, Antonelli MC. Prenatal stress and neurodevelopmental plasticity: relevance to psychopathology. Adv Exp Med Biol. 2017;1015:117–29. 29080024 10.1007/978-3-319-62817-2_7

[55] Baier CJ, Katunar MR, Adrover E, Pallares ME, Antonelli MC. Gestational restraint stress and the developing dopaminergic system: an overview. Neurotox Res. 2012;22:16–32. 22215534 10.1007/s12640-011-9305-4

[56] Weinstock M. Prenatal stressors in rodents: effects on behavior. Neurobiol Stress. 2017;6:3–13. 28229104 10.1016/j.ynstr.2016.08.004 PMC5314420

[57] Buynitsky T, Mostofsky DI. Restraint stress in biobehavioral research: recent developments. Neurosci Biobehav Rev. 2009;33:1089–98. 19463853 10.1016/j.neubiorev.2009.05.004

[58] Servatius RJ, Salameh G, Coyle KM, Paré WP. Restraint Stress. Encyclopedia of Stress2007. pp. 389 – 90.

[59] Atrooz F, Alkadhi KA, Salim S. Understanding stress: insights from rodent models. Curr Res Neurobiol. 2021;2. 10.1016/j.crneur.2021.100013 PMC9559100 36246514

[60] Truett GE, Heeger P, Mynatt RL, Truett AA, Walker JA, Warman ML. Preparation of PCR-quality mouse genomic DNA with hot sodium hydroxide and tris (HotSHOT). Biotechniques. 2000;29:52. 10907076 10.2144/00291bm09

[61] Dhakal P, Soares MJ. Single-step PCR-based genetic sex determination of rat tissues and cells. Biotechniques. 2017;62:232–3. 28528577 10.2144/000114548 PMC5831150

[62] Thery C, Amigorena S, Raposo G, Clayton A. Isolation and characterization of exosomes from cell culture supernatants and biological fluids. Curr Protoc Cell Biol. 2006;Chap. 3:Unit 3 22. 10.1002/0471143030.cb0322s30 18228490

[63] Li SX, Fujita Y, Zhang JC, Ren Q, Ishima T, Wu J, et al. Role of the NMDA receptor in cognitive deficits, anxiety and depressive-like behavior in juvenile and adult mice after neonatal dexamethasone exposure. Neurobiol Dis. 2014;62:124–34. 24051277 10.1016/j.nbd.2013.09.004

[64] Zhang WJ, Cao WY, Huang YQ, Cui YH, Tu BX, Wang LF, et al. The role of miR-150 in stress-Induced anxiety-like Behavior in mice. Neurotox Res. 2019;35:160–72. 30120712 10.1007/s12640-018-9943-x

[65] Yuen EY, Wei J, Liu W, Zhong P, Li X, Yan Z. Repeated stress causes cognitive impairment by suppressing glutamate receptor expression and function in prefrontal cortex. Neuron. 2012;73:962–77. 22405206 10.1016/j.neuron.2011.12.033 PMC3302010

[66] Thome J, Pesold B, Baader M, Hu M, Gewirtz JC, Duman RS, et al. Stress differentially regulates synaptophysin and synaptotagmin expression in hippocampus. Biol Psychiatry. 2001;50:809–12. 11720700 10.1016/s0006-3223(01)01229-x

[67] Xu H, He J, Richardson JS, Li XM. The response of synaptophysin and microtubule-associated protein 1 to restraint stress in rat hippocampus and its modulation by venlafaxine. J Neurochem. 2004;91:1380–8. 15584914 10.1111/j.1471-4159.2004.02827.x

[68] Levine J, Kwon E, Paez P, Yan W, Czerwieniec G, Loo JA, et al. Traumatically injured astrocytes release a proteomic signature modulated by STAT3-dependent cell survival. Glia. 2016;64:668–94. 26683444 10.1002/glia.22953 PMC4805454

[69] Fride E, Weinstock M. Prenatal stress increases anxiety related behavior and alters cerebral lateralization of dopamine activity. Life Sci. 1988;42:1059–65. 3347138 10.1016/0024-3205(88)90561-9

[70] Vallee M, Mayo W, Dellu F, Le Moal M, Simon H, Maccari S. Prenatal stress induces high anxiety and postnatal handling induces low anxiety in adult offspring: correlation with stress-induced corticosterone secretion. J Neurosci. 1997;17:2626–36. 9065522 10.1523/JNEUROSCI.17-07-02626.1997 PMC6573515

[71] Barros VG, Rodriguez P, Martijena ID, Perez A, Molina VA, Antonelli MC. Prenatal stress and early adoption effects on benzodiazepine receptors and anxiogenic behavior in the adult rat brain. Synapse. 2006;60:609–18. 17019679 10.1002/syn.20336

[72] Soares-Cunha C, Coimbra B, Borges S, Domingues AV, Silva D, Sousa N, et al. Mild prenatal stress causes emotional and brain structural modifications in rats of both sexes. Front Behav Neurosci. 2018;12:129. 30034328 10.3389/fnbeh.2018.00129 PMC6043801

[73] Morley-Fletcher S, Darnaudery M, Koehl M, Casolini P, Van Reeth O, Maccari S. Prenatal stress in rats predicts immobility behavior in the forced swim test. Effects of a chronic treatment with tianeptine. Brain Res. 2003;989:246–51. 14556947 10.1016/s0006-8993(03)03293-1

[74] Sun H, Guan L, Zhu Z, Li H. Reduced levels of NR1 and NR2A with depression-like behavior in different brain regions in prenatally stressed juvenile offspring. PLoS ONE. 2013;8:e81775. 24278457 10.1371/journal.pone.0081775 PMC3835745

[75] Lemaire V, Koehl M, Le Moal M, Abrous DN. Prenatal stress produces learning deficits associated with an inhibition of neurogenesis in the hippocampus. Proc Natl Acad Sci U S A. 2000;97:11032–7. 11005874 10.1073/pnas.97.20.11032 PMC27143

[76] Son GH, Geum D, Chung S, Kim EJ, Jo JH, Kim CM, et al. Maternal stress produces learning deficits associated with impairment of NMDA receptor-mediated synaptic plasticity. J Neurosci. 2006;26:3309–18. 16554481 10.1523/JNEUROSCI.3850-05.2006 PMC6674110

[77] Yang J, Han H, Cao J, Li L, Xu L. Prenatal stress modifies hippocampal synaptic plasticity and spatial learning in young rat offspring. Hippocampus. 2006;16:431–6. 16598704 10.1002/hipo.20181

[78] Gannouni N, Mhamdi A, El May M, Tebourbi O, Rhouma KB. Morphological changes of adrenal gland and heart tissue after varying duration of noise exposure in adult rat. Noise Health. 2014;16:416–21. 25387538 10.4103/1463-1741.144424

[79] Oliveira MJ, Monteiro MP, Ribeiro AM, Pignatelli D, Aguas AP. Chronic exposure of rats to occupational textile noise causes cytological changes in adrenal cortex. Noise Health. 2009;11:118–23. 19414932 10.4103/1463-1741.50697

[80] Zaki SM, Abdelgawad FA, El-Shaarawy EAA, Radwan RAK, Aboul-Hoda BE. Stress-induced changes in the aged-rat adrenal cortex. Histological and histomorphometric study. Folia Morphol (Warsz). 2018;77:629–41. 29611160 10.5603/FM.a2018.0035

[81] Sarapultsev PA, Chupakhin ON, Medvedeva SU, Mukhlynina EA, Brilliant SA, Sidorova LP, et al. The impact of immunomodulator compound from the group of substituted thiadiazines on the course of stress reaction. Int Immunopharmacol. 2015;25:440–9. 25737199 10.1016/j.intimp.2015.02.024

[82] Diaz-Aguila Y, Cuevas-Romero E, Castelan F, Martinez-Gomez M, Rodriguez-Antolin J, Nicolas-Toledo L. Chronic stress and high sucrose intake cause distinctive morphometric effects in the adrenal glands of post-weaned rats. Biotech Histochem. 2018;93:565–74. 30136861 10.1080/10520295.2018.1499961

[83] Jeong JY, Lee DH, Kang SS. Effects of chronic restraint stress on body weight, food intake, and hypothalamic gene expressions in mice. Endocrinol Metab (Seoul). 2013;28:288–96. 24396694 10.3803/EnM.2013.28.4.288 PMC3871039

[84] Lee YE, Byun SK, Shin S, Jang JY, Choi BI, Park D, et al. Effect of maternal restraint stress on fetal development of ICR mice. Exp Anim. 2008;57:19–25. 18256515 10.1538/expanim.57.19

[85] Trifunovic S, Sosic Jurjevic B, Ristic N, Nestorovic N, Filipovic B, Stevanovic I et al. Maternal dexamethasone exposure induces sex-specific changes in Histomorphology and Redox Homeostasis of Rat Placenta. Int J Mol Sci. 2022;24. 10.3390/ijms24010540 PMC9820254 36613982

[86] Ellman LM, Murphy SK, Maxwell SD, Calvo EM, Cooper T, Schaefer CA, et al. Maternal cortisol during pregnancy and offspring schizophrenia: influence of fetal sex and timing of exposure. Schizophr Res. 2019;213:15–22. 31345704 10.1016/j.schres.2019.07.002 PMC7074891

[87] Ellman LM, Schetter CD, Hobel CJ, Chicz-Demet A, Glynn LM, Sandman CA. Timing of fetal exposure to stress hormones: effects on newborn physical and neuromuscular maturation. Dev Psychobiol. 2008;50:232–41. 18335490 10.1002/dev.20293 PMC2851937

[88] Class QA, Lichtenstein P, Langstrom N, D’Onofrio BM. Timing of prenatal maternal exposure to severe life events and adverse pregnancy outcomes: a population study of 2.6 million pregnancies. Psychosom Med. 2011;73:234–41. 21321257 10.1097/PSY.0b013e31820a62ce PMC3070756

[89] Rosenfeld CS. Sex-specific placental responses in fetal development. Endocrinology. 2015;156:3422–34. 26241064 10.1210/en.2015-1227 PMC4588817

[90] Nugent BM, Bale TL. The omniscient placenta: metabolic and epigenetic regulation of fetal programming. Front Neuroendocrinol. 2015;39:28–37. 26368654 10.1016/j.yfrne.2015.09.001 PMC4681645

[91] Gabory A, Roseboom TJ, Moore T, Moore LG, Junien C. Placental contribution to the origins of sexual dimorphism in health and diseases: sex chromosomes and epigenetics. Biol Sex Differ. 2013;4:5. 23514128 10.1186/2042-6410-4-5 PMC3618244

[92] Howerton CL, Bale TL. Targeted placental deletion of OGT recapitulates the prenatal stress phenotype including hypothalamic mitochondrial dysfunction. Proc Natl Acad Sci U S A. 2014;111:9639–44. 24979775 10.1073/pnas.1401203111 PMC4084439

[93] Howerton CL, Morgan CP, Fischer DB, Bale TL. O-GlcNAc transferase (OGT) as a placental biomarker of maternal stress and reprogramming of CNS gene transcription in development. Proc Natl Acad Sci U S A. 2013;110:5169–74. 23487789 10.1073/pnas.1300065110 PMC3612602

[94] Woods L, Perez-Garcia V, Hemberger M. Regulation of placental development and its impact on fetal growth-new insights from mouse models. Front Endocrinol (Lausanne). 2018;9:570. 30319550 10.3389/fendo.2018.00570 PMC6170611

[95] Myatt L. Placental adaptive responses and fetal programming. J Physiol. 2006;572:25–30. 16469781 10.1113/jphysiol.2006.104968 PMC1779654

[96] Fowden AL, Forhead AJ, Coan PM, Burton GJ. The placenta and intrauterine programming. J Neuroendocrinol. 2008;20:439–50. 18266944 10.1111/j.1365-2826.2008.01663.x

[97] Rossant J, Cross JC. Placental development: lessons from mouse mutants. Nat Rev Genet. 2001;2:538–48. 11433360 10.1038/35080570

[98] Buckberry S, Bianco-Miotto T, Bent SJ, Dekker GA, Roberts CT. Integrative transcriptome meta-analysis reveals widespread sex-biased gene expression at the human fetal-maternal interface. Mol Hum Reprod. 2014;20:810–9. 24867328 10.1093/molehr/gau035 PMC4106635

[99] Mao J, Zhang X, Sieli PT, Falduto MT, Torres KE, Rosenfeld CS. Contrasting effects of different maternal diets on sexually dimorphic gene expression in the murine placenta. Proc Natl Acad Sci U S A. 2010;107:5557–62. 20212133 10.1073/pnas.1000440107 PMC2851754

[100] O’Connell BA, Moritz KM, Walker DW, Dickinson H. Sexually dimorphic placental development throughout gestation in the spiny mouse (Acomys cahirinus). Placenta. 2013;34:119–26. 23260227 10.1016/j.placenta.2012.11.009

[101] Sood R, Zehnder JL, Druzin ML, Brown PO. Gene expression patterns in human placenta. Proc Natl Acad Sci U S A. 2006;103:5478–83. 16567644 10.1073/pnas.0508035103 PMC1414632

[102] Bronson SL, Bale TL. Prenatal stress-induced increases in placental inflammation and offspring hyperactivity are male-specific and ameliorated by maternal antiinflammatory treatment. Endocrinology. 2014;155:2635–46. 24797632 10.1210/en.2014-1040 PMC4060181

[103] Mairesse J, Lesage J, Breton C, Breant B, Hahn T, Darnaudery M, et al. Maternal stress alters endocrine function of the feto-placental unit in rats. Am J Physiol Endocrinol Metab. 2007;292:E1526–33. 17264224 10.1152/ajpendo.00574.2006

[104] Briffa JF, Hosseini SS, Tran M, Moritz KM, Cuffe JSM, Wlodek ME. Maternal growth restriction and stress exposure in rats differentially alters expression of components of the placental glucocorticoid barrier and nutrient transporters. Placenta. 2017;59:30–8. 29108634 10.1016/j.placenta.2017.09.006

[105] Jensen Pena C, Monk C, Champagne FA. Epigenetic effects of prenatal stress on 11beta-hydroxysteroid dehydrogenase-2 in the placenta and fetal brain. PLoS ONE. 2012;7:e39791. 22761903 10.1371/journal.pone.0039791 PMC3383683

[106] Napso T, Hung YP, Davidge ST, Care AS, Sferruzzi-Perri AN. Advanced maternal age compromises fetal growth and induces sex-specific changes in placental phenotype in rats. Sci Rep. 2019;9:16916. 31780670 10.1038/s41598-019-53199-x PMC6882885

[107] Elser BA, Kayali K, Dhakal R, O’Hare B, Wang K, Lehmler HJ, et al. Combined maternal exposure to Cypermethrin and stress affect embryonic brain and placental outcomes in mice. Toxicol Sci. 2020;175:182–96. 32191333 10.1093/toxsci/kfaa040 PMC7253216

[108] Cuffe JS, O’Sullivan L, Simmons DG, Anderson ST, Moritz KM. Maternal corticosterone exposure in the mouse has sex-specific effects on placental growth and mRNA expression. Endocrinology. 2012;153:5500–11. 22919064 10.1210/en.2012-1479

[109] Ge Y, Wei M, Chang X, Huang Y, Duan T, Wang K, et al. Alterations in maternal plasma exosomal miRNAs revealed selective material exchange between maternal circulation and placenta. J Obstet Gynaecol Res. 2023;49:109–21. 36216398 10.1111/jog.15452

[110] Foley HB, Howe CG, Eckel SP, Chavez T, Gevorkian L, Reyes EG, et al. Extracellular vesicle-enriched miRNA profiles across pregnancy in the MADRES cohort. PLoS ONE. 2021;16:e0251259. 33979365 10.1371/journal.pone.0251259 PMC8115775

[111] Salomon C, Rice GE. Role of exosomes in placental homeostasis and pregnancy disorders. Prog Mol Biol Transl Sci. 2017;145:163–79. 28110750 10.1016/bs.pmbts.2016.12.006

[112] Mitchell MD, Peiris HN, Kobayashi M, Koh YQ, Duncombe G, Illanes SE, et al. Placental exosomes in normal and complicated pregnancy. Am J Obstet Gynecol. 2015;213:S173–81. 26428497 10.1016/j.ajog.2015.07.001

[113] Miranda J, Paules C, Nair S, Lai A, Palma C, Scholz-Romero K, et al. Placental exosomes profile in maternal and fetal circulation in intrauterine growth restriction - liquid biopsies to monitoring fetal growth. Placenta. 2018;64:34–43. 29626979 10.1016/j.placenta.2018.02.006

[114] Nair S, Salomon C. Extracellular vesicles and their immunomodulatory functions in pregnancy. Semin Immunopathol. 2018;40:425–37. 29616307 10.1007/s00281-018-0680-2

[115] Edgar JR, Manna PT, Nishimura S, Banting G, Robinson MS. Tetherin is an exosomal tether. Elife. 2016;5. 10.7554/eLife.17180 PMC5033606 27657169

[116] Mateescu B, Kowal EJ, van Balkom BW, Bartel S, Bhattacharyya SN, Buzas EI, et al. Obstacles and opportunities in the functional analysis of extracellular vesicle RNA - an ISEV position paper. J Extracell Vesicles. 2017;6:1286095. 28326170 10.1080/20013078.2017.1286095 PMC5345583

[117] Colombo M, Raposo G, Thery C. Biogenesis, secretion, and intercellular interactions of exosomes and other extracellular vesicles. Annu Rev Cell Dev Biol. 2014;30:255–89. 25288114 10.1146/annurev-cellbio-101512-122326

[118] Yang C, Song G, Lim W. Effects of extracellular vesicles on placentation and pregnancy disorders. Reproduction. 2019;158:R189–96. 31247586 10.1530/REP-19-0147

[119] Zhang J, Li H, Fan B, Xu W, Zhang X. Extracellular vesicles in normal pregnancy and pregnancy-related diseases. J Cell Mol Med. 2020;24:4377–88. 32175696 10.1111/jcmm.15144 PMC7176865

[120] Dickens AM, Tovar YRLB, Yoo SW, Trout AL, Bae M, Kanmogne M et al. Astrocyte-shed extracellular vesicles regulate the peripheral leukocyte response to inflammatory brain lesions. Sci Signal. 2017;10. 10.1126/scisignal.aai7696 PMC5590230 28377412

[121] Cai S, Shi GS, Cheng HY, Zeng YN, Li G, Zhang M, et al. Exosomal miR-7 mediates Bystander Autophagy in Lung after Focal Brain Irradiation in mice. Int J Biol Sci. 2017;13:1287–96. 29104495 10.7150/ijbs.18890 PMC5666527

[122] Arnsten AF. Stress signalling pathways that impair prefrontal cortex structure and function. Nat Rev Neurosci. 2009;10:410–22. 19455173 10.1038/nrn2648 PMC2907136

[123] Duman RS, Aghajanian GK, Sanacora G, Krystal JH. Synaptic plasticity and depression: new insights from stress and rapid-acting antidepressants. Nat Med. 2016;22:238–49. 26937618 10.1038/nm.4050 PMC5405628

[124] Lafourcade CA, Fernandez A, Ramirez JP, Corvalan K, Carrasco MA, Iturriaga A et al. A role for mir-26a in stress: a potential sEV biomarker and modulator of excitatory neurotransmission. Cells. 2020;9. 10.3390/cells9061364 PMC7349773 32492799

