# Supplementary figures and images for: Maternal stress during pregnancy alters circulating small extracellular vesicles and enhances their targeting to the placenta and fetus
Source: Biol Res. 2024 Sep 28;57:70. doi: 10.1186/s40659-024-00548-4 (PMC11438166; doi:10.1186/s40659-024-00548-4)

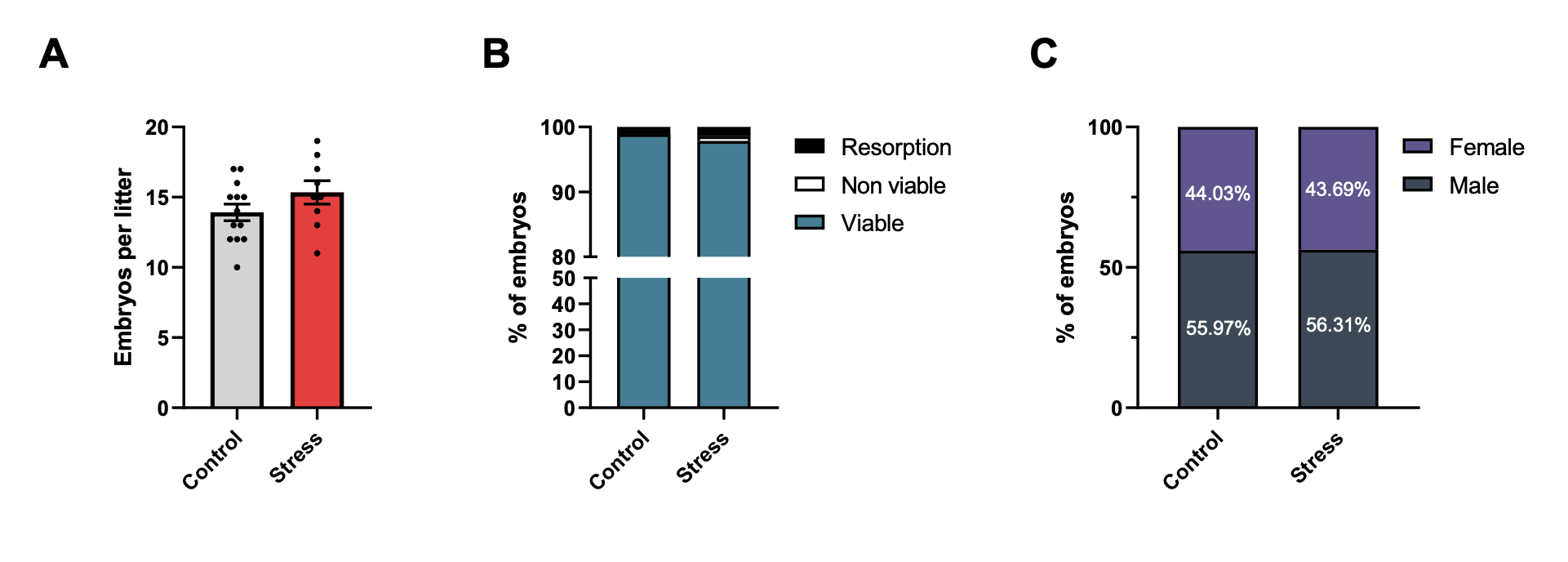

Supplement: Supplementary file 1 — Supplementary Material 1 [file 40659_2024_548_MOESM1_ESM.tiff]

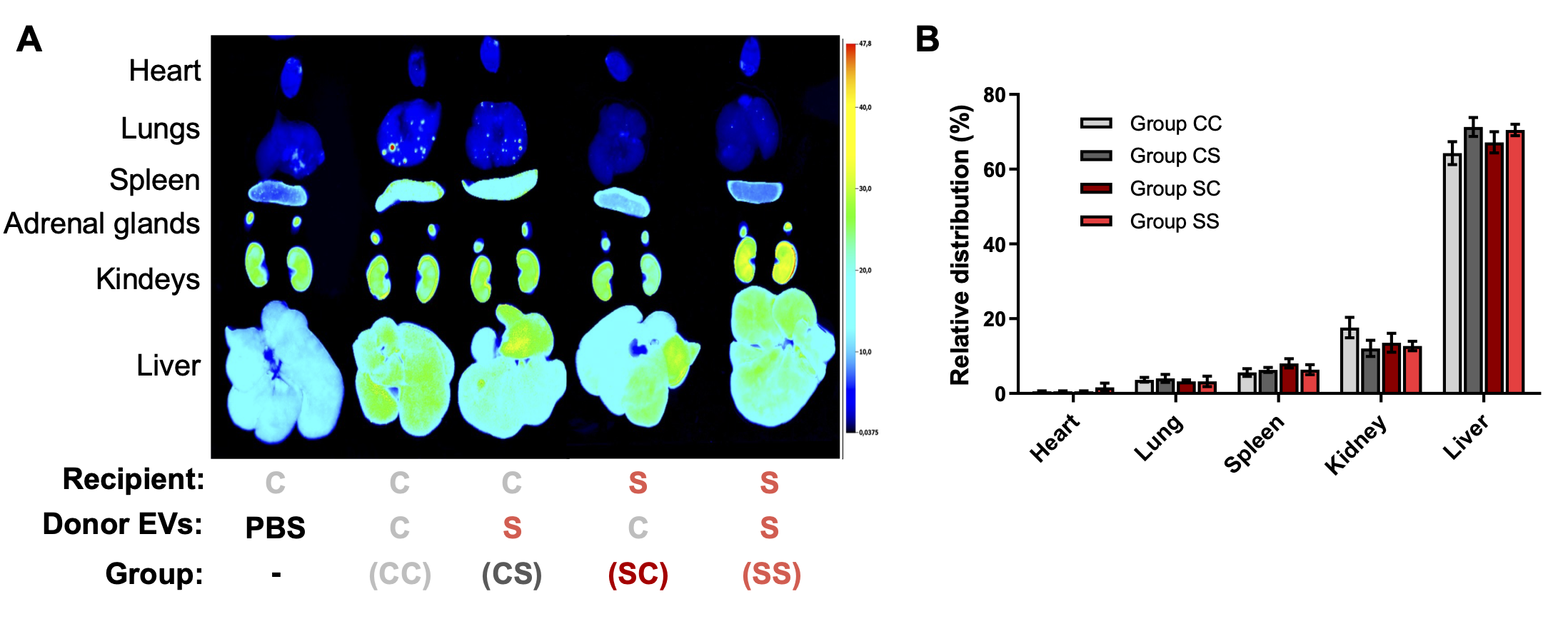

Supplement: Supplementary file 2 — Supplementary Material 2 [file 40659_2024_548_MOESM2_ESM.tiff]
